# Dementia, gait disturbance, and urinary incontinence in a patient with pulmonary sarcoidosis

**DOI:** 10.1002/rcr2.182

**Published:** 2016-08-26

**Authors:** Gonzalo Labarca, Romina Ramirez, Ximena Monsalve, Isabel Mira‐Avendano

**Affiliations:** ^1^ Department of Internal MedicineComplejo Asistencial Victor Rios RuizLos ÁngelesChile; ^2^ Department of Internal MedicinePontifical Catholic UniversitySantiagoChile; ^3^ Department of Internal MedicineHospital Sotero del RíoSantiagoChile; ^4^ Department of Pulmonary MedicineMayo ClinicJacksonvilleFloridaUSA

**Keywords:** Sarcoidosis, dementia, neurosarcoidosis, hydrocephalus

## Abstract

Hydrocephalus is an uncommon presentation of neurosarcoidosis. We discuss the case of a 67‐year‐old woman with a prior diagnosis of hypothyroidism, systemic hypertension, and lung sarcoidosis who presented with a 1‐month history of progressive impairment of consciousness leading to prostration and loss of sphincter control. At admission, patient was febrile with altered speech and without focalization. Laboratory results showed leucocytosis and mild anaemia. Cerebrospinal fluid (CSF) analysis was characterized by mild elevated protein level, increased cell count, normal glucose and adenosine deaminase (ADA), negative cytology, and no bacterial isolations. Electroencephalogram showed toxic‐metabolic encephalopathy. Computed tomography (CT) of the brain revealed hydrocephalus without structural damage, and magnetic resonance imaging of the brain demonstrated non‐specific diffuse meningeal enhancement and periventricular changes supporting normal pressure hydrocephalus. Chest X‐ray showed mediastinal adenopathy and parenchymatous lesions, consistent with stage II lung sarcoidosis. A ventriculoperitoneal shunt was installed, and the patient experienced rapid improvement in her symptoms.

## Introduction

Sarcoidosis is a multisystem syndrome of unknown aetiology, characterized by the formation of multiple non‐caseating granulomas that alter the affected tissue. There are no Chilean studies of prevalence. In North America, incidences of 35.5 cases per 1,000,000 in African American and 10.9 cases per 1,000,000 in Caucasian populations have been reported. The lungs are affected in approximately 90% of patients. Other commonly involved tissues include the skin, eyes, and lymph nodes 1, 2. Only 5%–10% of patients with sarcoidosis suffer neurological involvement, with positive symptomatology present in half of them 2.

We report the case of a woman with a prior diagnosis of lung sarcoidosis who developed rapidly progressing dementia, secondary to normal pressure hydrocephalus (NPH), explained by neurological involvement in this disease.

## Case Report

**Figure 1:**
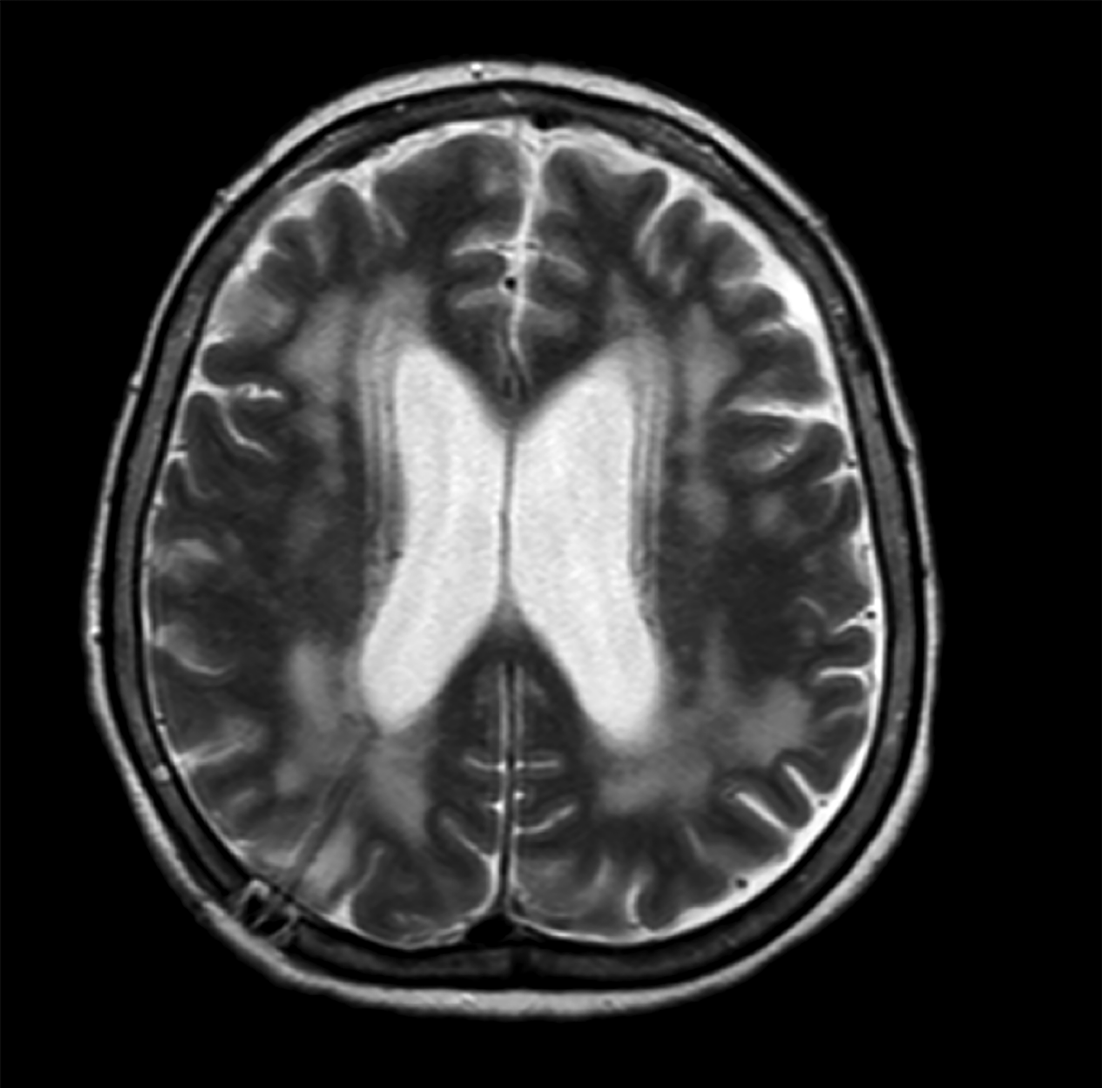
Magnetic resonance imaging revealing quadriventricular hydrocephalus, non‐specific periventricular change, and diffuse meningeal enhancement.

**Figure 2:**
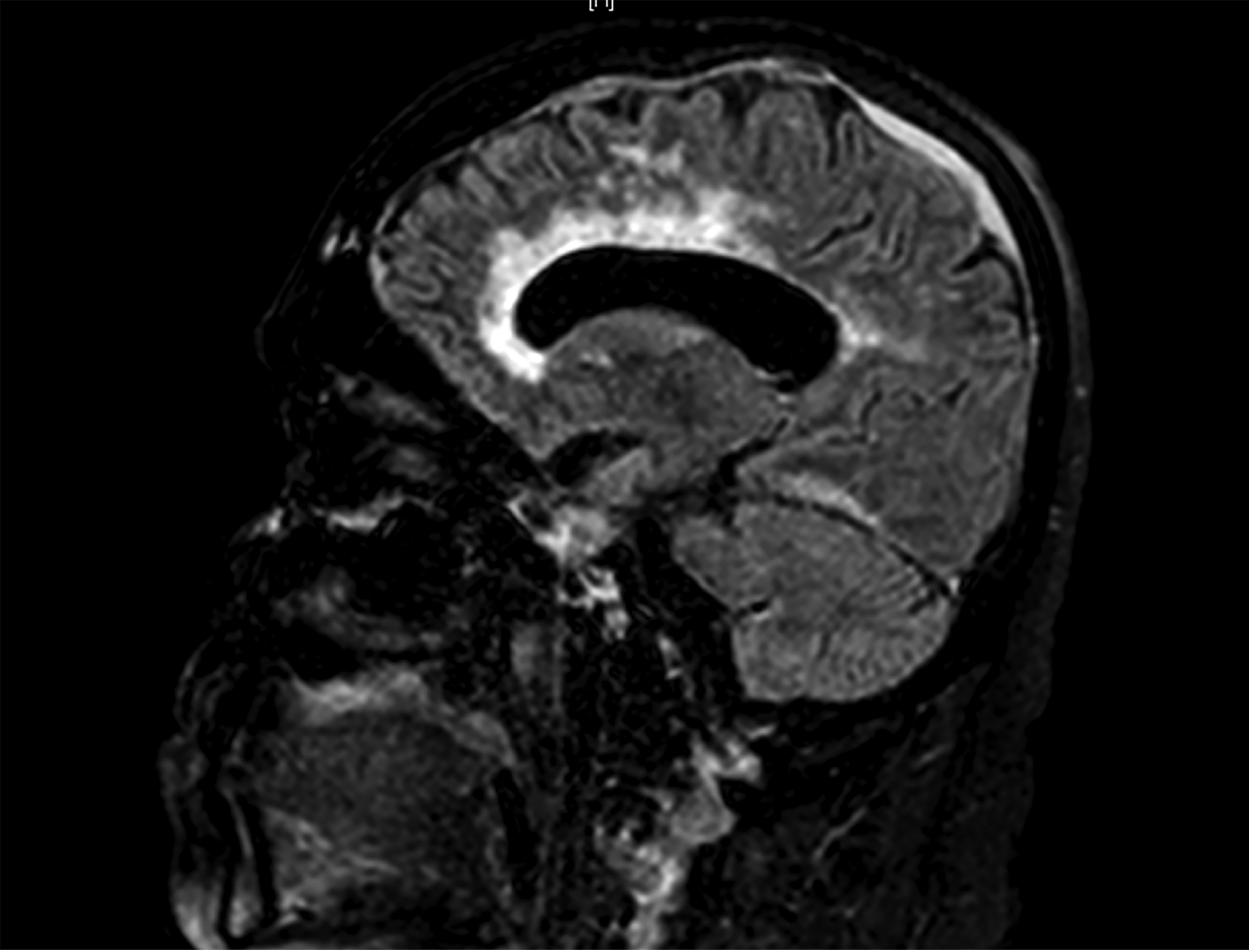
Magnetic resonance imaging revealing diffuse leucoencephalopathy and meningeal enhancement (occipital predominance).

A 67‐year‐old Hispanic woman with a past medical history of hypothyroidism, hypertension, and recently diagnosed stage II pulmonary sarcoidosis (confirmed through transbronchial biopsy 2 months prior) presented with a 1‐month history of progressive impairment of consciousness that was associated with decreased strength and progressed to prostration and loss of sphincter control. At initial evaluation in the emergency department, she was found to be haemodynamically stable, febrile (39°C), somnolent, with non‐comprehensible speech, and with no signs of neurological focalization. The rest of her physical examination was unremarkable. Cerebrospinal fluid (CSF) showed protein levels of 0.66 g/L (normal value < 0.5 g/L), 70 cells (mostly mononuclear), glucose levels of 45 mg/dL (normal value > 60 mg/dL), and adenosine deaminase (ADA) levels of 3.3 U/L (normal value < 7 U/L). Gram stain was negative for bacteria and the bacterial and fungus cultures were negative. Computed tomography (CT) of the brain revealed hydrocephalus without structural lesions. The study was complemented with magnetic resonance imaging (MRI) that revealed non‐specific periventricular changes and diffuse meningeal enhancement, quadriventricular hydrocephalus, and diffuse leucoencephalopathy. The image was compatible with NPH (Figs. 1, 2).

**Figure 3:**
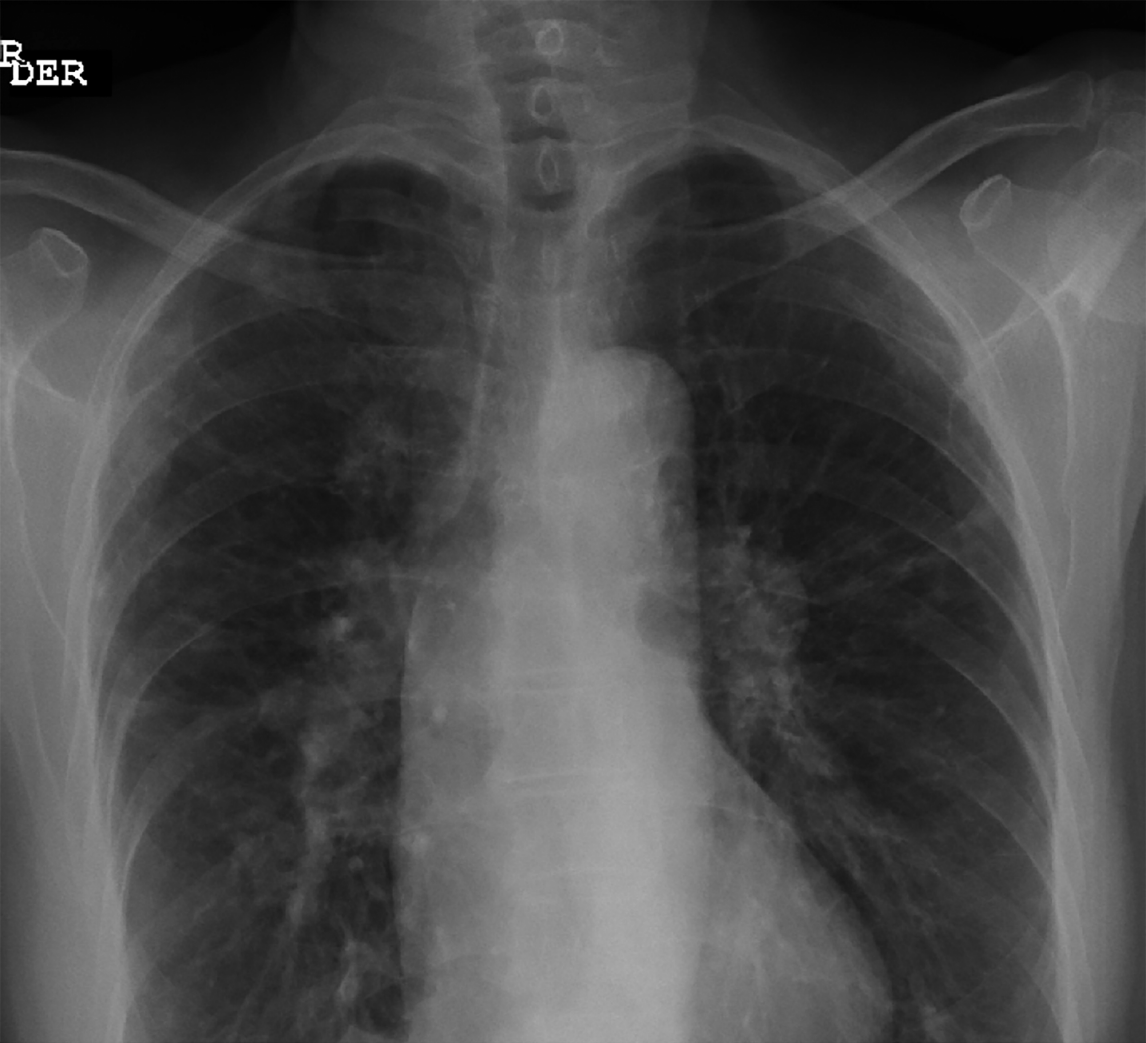
Chest X‐ray showing pulmonary sarcoidosis.

Complementary studies, including HIV and venereal disease research laboratory (VDRL) tests, were negative. Electroencephalogram (EEG) showed non‐specific changes suggesting diffuse encephalopathy due to metabolic abnormalities, and the patient had normal thyroid panel, complement and B12 levels, with negative connective tissue serologies. Chest X‐ray showed grade II pulmonary sarcoidosis (Fig. 3).

After a multidisciplinary evaluation, a ventriculoperitoneal (VP) shunt was installed without complications, and the patient was started on steroid therapy with prednisone of 0.5 mg per kg, with rapid improvement in her symptoms. After 2 years of follow‐up, patient recovery was successful, without fever or evidence of infection. Current medication includes prednisone of 5 mg per day and azathioprine of 100 mg per day.

## Discussion

We report an unusual cause of neurosarcoidosis in association with pulmonary involvement. Neurosarcoidosis is a severe disease condition and the most common manifestation is cranial nerve compromise, most often presenting as peripheral seventh nerve palsy. Other possible manifestations are hydrocephalus, myopathy, peripheral neuropathy, meningitis, and seizures 3, 4, 5, 6, 7. But the rates of asymptomatic involvement may be higher, with positive autopsy findings in up to 15% of patients 8. When neurological manifestations occur, it usually happens during the first two years of diagnosis, corresponding to isolated manifestation of the disease in only 1% of the cases 1, 9, and three‐fifths of neurosarcoidosis cases are characterized by lesions at different levels (brain, leptomeningeal, or peripheral nerve) 3, 10, 11, 12. A summary of clinical manifestations of neurosarcoidosis is shown in Table 1.

**Table 1:** Neurological involvement in sarcoidosis.

	Frequency	Clinical finding	Comments
Cranial neuropathies	25%–50%	Unilateral facial palsy Optic neuropathy Eight nerve palsy Multiple cranial nerve	Bilateral neuropathy, bad prognosis. Bilateral eight neuropathy involvement is highly suggestive of neurosarcoidosis.
Meningeal involvement	40%	Aseptic meningitis Chronic meningitis (>4 weeks) Hydrocephalus	Treatment includes steroids and prognosis is good. Treatment for chronic meningitis includes long‐term therapy.
Granulomatous involvement		Partial or generalized seizures Encephalopathy/vasculopathy Focal cerebral infarction	Patients can present with cognitive or behavioural problems and/or focal neurological deficits referable to the anatomic area involved.
Neuroendocrine dysfunction		Hypothalamic dysfunction Diabetes insipidus Adenopituitary failure Amenorrhea‐galactorrhea syndrome	Polyuria and polydipsia are the more common presentations, due to either diabetes insipidus or disturbances of thirst. Other clinical symptoms include disorders in sleep, appetite, temperature, or libido.
Myelopathy/radiculopathy	16%–43%	Spinal cord compression Mononeuritis multiplex Caudal equine Guillain–Barre syndrome Longitudinally extensive myelitis	Clinical involvement includes paraesthesia, muscular weakness, and paraplegia of lower limbs in patients with severe disease.

Our patient developed hydrocephalus, an uncommon presentation of neurosarcoidosis, with few previously reported cases 7, 13. This manifestation is likely secondary to pathological granulation of arachnoids. Normal pressure hydrocephalus refers to a ventricular enlargement with normal opening pressures on lumbar puncture. Normal pressure hydrocephalus is characterized by the classic triad of dementia, gait disturbance, and urinary incontinence. Prognosis in these cases is poor, with mortality within 1 year for about 75% of patients 8, 14 and it is important to recognize these cases because they can be reversed by the placement of a VP shunt. The most probable mechanism in these cases is the impaired absorption of CSF secondary to chronic granuloma or pachymeningitis.

Laboratory evaluations in neurosarcoidosis include general exams and lumbar puncture. Although they have low diagnosis yields, lumbar puncture and CSF analysis should always be considered because some abnormalities such as elevated immunoglobulin (Ig)G index, oligoclonal bands, and high levels of elevated angiotensin enzyme can aid in the diagnosis 8. In addition, elevated protein levels and CSF opening pressure, along with pleocytosis (predominantly mononuclear cell), are characteristic of NPH.

In regard to neuroimaging, MRI is considered the most sensitive non‐invasive test for neurosarcoidosis 5, but a normal result does not exclude the diagnosis. The most common MRI finding is leptomeningeal involvement, with nodules or plaques seen when contrast is used 8, 15. Damage to the spinal cord, cauda equina, and the cranial nerves can also be seen. In a series of 30 cases in patients diagnosed with neurosarcoidosis, 40% presented with positive MRI findings, such as meningeal enhancement and/or multiple white matter lesions 16. Our patient presented a hydrocephalus and diffuse meningeal enhancement, changes suggestive of neurosarcoidosis.

Finally, pathology showing granulomatous lesions is the most specific diagnostic test for sarcoidosis, but because biopsy of peripheral or central nerves carries high morbidity, it is usually supported with samples taken from other organs, particularly the lungs and lymph nodes 1, 8, 14, 17. We considered the clinical manifestation in association with imaging and differential diagnosis ruled out, in combination with a known history of pulmonary sarcoidosis as diagnostic in this case, and no pathological sample was needed in order to treat the patient.

In most cases, immunosuppressive therapy is needed for the control of neurosarcoidosis. Corticosteroids remain the first‐line treatment 8, 11, 17.

Most patients respond to treatment and are able to tolerate steroid withdrawal after several months, although stronger immunosuppressants such as methotrexate, azathioprine, cyclophosphamide, cyclosporine, and more recently infliximab have also been used 17, 18. In some cases, such as ours, surgical intervention is indicated.

This case shows an uncommon clinical presentation of neurosarcoidosis with rapid progressive loss of consciousness secondary to hydrocephalus and emphasizes the importance of a timely diagnosis to ensure a good prognosis for the patient.

## Disclosure Statements

No conflict of interest declared.

Appropriate written informed consent was obtained for publication of this case report and accompanying images.
